# Whole genome sequencing revealed esophageal squamous cell carcinoma related biomarkers

**DOI:** 10.1371/journal.pone.0323915

**Published:** 2025-06-26

**Authors:** Mingjun Li, Lei Li, Xizi Wang, Yanwei Zhao, Peina Du, Wei Wang, Zhenxing Wang, Yadong Wang, Yanxing Sheng, Mingliang Gu, Xiaodong Jia

**Affiliations:** 1 Department of Radiotherapy, Liaocheng People’s Hospital, Liaocheng, Shandong, China; 2 BGI Research, Shenzhen, China; 3 BGI Research, Qingdao, China; 4 Joint Laboratory for Translational Medicine Research, Liaocheng People’s Hospital, Liaocheng, Shandong, China; 5 BGI Genomics, Shenzhen, China; 6 Clin Lab, BGI Genomics, Qingdao, China; 7 Department of Thoracic Surgery, Liaocheng People’s Hospital, Liaocheng, Shandong, China; Shantou University Medical College, CHINA

## Abstract

Esophageal squamous cell carcinoma (ESCC) is among the most frequently diagnosed cancer types, and affected patients frequently experience poor prognostic outcomes and high mortality rates. Many genomic studies of ESCC have been performed in recent years, yet the mutational mechanisms driving ESCC and their clinical implications remain incompletely understood. In this study, paired tumor and normal tissue samples from 22 patients with ESCC were used for whole genome sequencing-based analyses of genome-wide mutational events. These comprehensive analyses enabled the detection and characterization of various mutation subtypes in ESCC including somatic single-nucleotide variants, small insertions and deletions, copy number variations, structural variations, and circular extrachromosomal DNA. Of identified genes harboring non-silent mutations, *TP53*, *NOTCH1*, *CSMD3*, *EP300*, and *FAM135B* were the most frequently mutated genes in this study and they were annotated in the COSMIC Cancer Gene Census. With the exception of aging-related signatures, an APOBEC-associated mutational signature was the dominant mutational feature detected in ESCC samples, suggesting that APOBEC-mediated cytidine deamination is likely a major driver of mutations in this cancer type. Notably, our study also detected circular extrachromosomal DNA (ecDNA) events in these ESCC patient samples. The oncogenes *COX6C*, *PVT1*, and *MMP12* as well as the oncogenic long non-coding RNA *AZIN1-AS1* which were detected in ecDNA regions in these analyses may be associated with worse disease-free survival in ESCC patients.

## Introduction

Esophageal cancer (EC) is the seventh most prevalent cancer type in the world, and over 90% of EC patients are diagnosed with esophageal squamous cell carcinoma (ESCC), which is a highly aggressive malignancy that is particularly common in China [1]. Within China, the incidence of ESCC exhibits marked geographic variability, with the highest rates in Taihang Mountain, Xinjiang province, and Chaoshan district, while incidence rates in other regions are rising rapidly [2]. At the global level, ESCC primarily impacts individuals in low- and middle-income nations, suggesting that exogenous exposures may contribute to the risk of ESCC development [3].

A range of lifestyle and environmental factors have been linked to the risk of ESCC [4]. There is strong evidence that tobacco use and alcohol abuse can synergistically contribute to ESCC incidence in lower-risk areas [4,5], while possible risk factors in China may include dietary habits including the consumption of hot food and betel nut chewing [6]. ESCC risk has also been linked to environmental factors including exposure to polycyclic aromatic hydrocarbons. While risk factors tied to the development of ESCC are increasingly well understood, these factors alone are not sufficient to account for the regional and national variations in ESCC incidence that have been observed to date [3].

ESCC is a tumor type characterized by poor prognostic outcomes owing to the limited clinical tools available for its early diagnosis [7]. Given its high degree of heterogeneity and the fact that clinical outcomes in patients tend to be variable, there are also few reliable prognostic biomarkers available for ESCC [8]. Understanding of the molecular mechanisms that govern ESCC progression and development is also relatively limited, underscoring the need to further define oncogenic changes and diagnostic biomarkers associated with the development of the devastating cancer type.

In this study, whole-genome sequencing (WGS) was performed for tumors and paired normal tissue samples from 22 patients with ESCC for whom corresponding clinical information was available. The resultant genomic data were analyzed to detect ESCC-related somatic mutations, copy number alterations (CNAs), and structural variations (SVs). In addition, key mutational signatures were analyzed, and circular extrachromosomal DNA (ecDNA)-related genes were examined in an effort to shed further light on the underlying molecular drivers of ESCC development.

## Results

### Patient characteristics

This study enrolled 22 ESCC patients (17 male, 5 female). Detailed clinical characteristics for these patients are summarized in S1 Table. Of these patients, 9 (40.9%) had a history of alcohol abuse and 12 (54.5%) had a history of smoking. Additionally, 1, 6, 12, and 3 of these patients were diagnosed with stage I, II, III, and IV tumors, respectively, and 16, 1, 4, and 1 patients were classified as having moderately differentiated, moderately or poorly differentiated, poorly differentiated, and 1 well differentiated tumors, respectively. The distribution of ESCC tumor locations in this study included upper thoracic (n = 1), middle thoracic (n = 9), and lower thoracic (n = 12) tumors (S1 Table).

**Fig 1:**
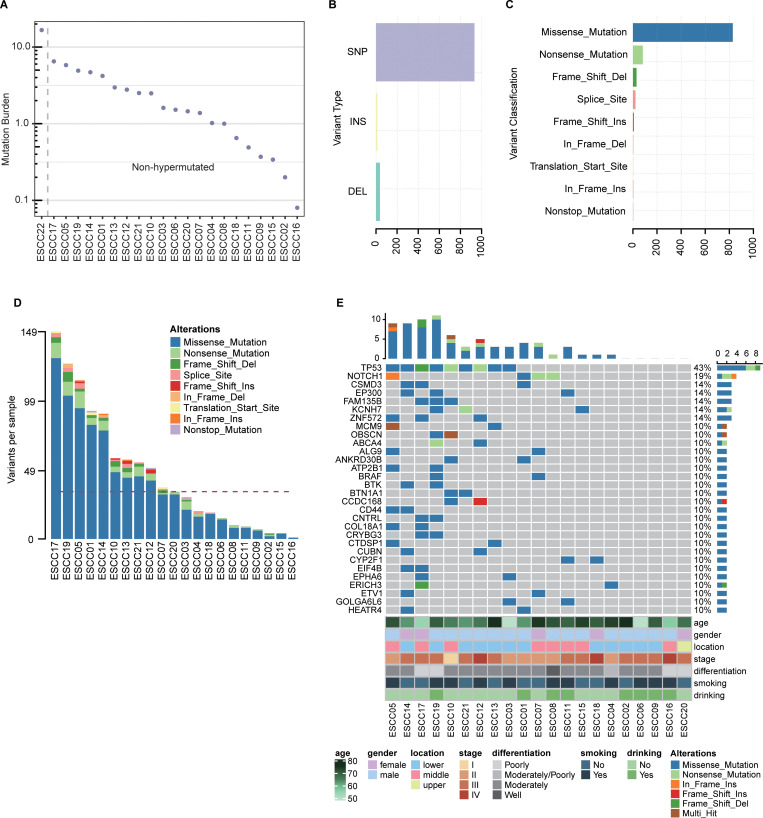
An overview of the somatic mutational profile of ESCC. (A) Somatic mutations per megabase of analyzed genomic sequence (mutation burden) in 22 ESCC patient tumor tissue samples. (B, C) Types (B) and classifications (C) of non-silent variants in non-hypermutated ESCC patients. (D) Non-silent Variants per sample in non-hypermutated ESCC patients. (E) Oncoplot for the top 30 mutated genes identified in patients with non-hypermutated ESCC.

WGS was performed for 22 tumors and matched normal tissues from ESCC patients, with respective average sequencing depths of 44.09x and 42.55x. Among these patients, one patient (ESCC22) was identified as having hypermutated ESCC, as evidenced by the presence of > 10 somatic mutations per megabase of analyzed genomic sequence (mutation burdern) (Fig 1A). This hypermutated patient was not included in subsequent analyses.

### The mutational landscape of ESCC

Short somatic mutations are among the most common mutation types, and were detected using Mutect2 in the present study. In total, 151,148 short somatic mutations were identified in these non-hypermutated ESCC patient samples, including 136,763 single nucleotide variants (SNVs), 948 multiple nucleotide polymorphisms (MNPs), and 13,437 insertions/deletions (INDELs) (4,060 insertions, 9,377 deletions) (S2 Table). Of these mutations, 1,304 were located in exonic or splicing regions (311 silent variants, 982 non-silent variants, and 11 unknown). The results of the 982 non-silent variants were summarized and visualized by R maftools and ComplexHeatmap packages (Fig 1B - E). There is an average of 47 non-silent mutations per tumor sample (range: 1–149) including 932 SNVs, 13 insertions, and 37 deletions (Fig 1B, D). And these somatic mutations were functionally annotated into missense mutations, nonsense mutations, frameshift deletions, splice site variants, frameshift insertions, in-frame deletions, translation start site (start-loss mutations), in-frame insertions, and nonstop mutations (stop-loss mutations) (Fig 1C). The mutated genes that were altered in at least 2 samples were mostly enriched in WNT and Notch signaling pathway using gene set enrichment analysis (S3 Table). In order to evaluate the protein interactive relationships among these genes, a PPI network was constructed (S1 Fig), and nodes that showed high scores in the network were screened as hub genes. Following STRING analysis and the MCC score, *TP53*, *PIK3CA*, and *NOTCH1* were found to play important roles. The most frequently mutated genes that were altered in at least three ESCC patient tumor samples included *TP53*, *NOTCH1*, *CSMD3*, *EP300*, *FAM135B*, *KCNH7*, and *ZNF572* (Fig 1E).

Notably, of these genes, *TP53*, *NOTCH1*, *CSMD3*, *EP300*, and *FAM135B* were all annotated in the COSMIC Cancer Gene Census, and the majority were impacted by missense or nonsense mutations in these ESCC patient samples (Fig 1E). The gene expressions of *TP53*, *NOTCH1*, *EP300* and *FAM135B* in primary tumor were different from solid tissue normal in The Cancer Genome Atlas Esophageal Carcinoma (TCGA-ESCA) cohort (S2 Fig, p < 0.05). And *TP53*, *CSMD3* and *NOTCH1* were also the most frequently mutated in TCGA-ESCA dataset (S3A Fig). The mutation burden identified by our study was lower than data from TCGA-ESCA (S3B Fig), probably due to the difference of sequencing method and sample size.

### Identification of ESCC-related mutational signatures

**Fig 2:**
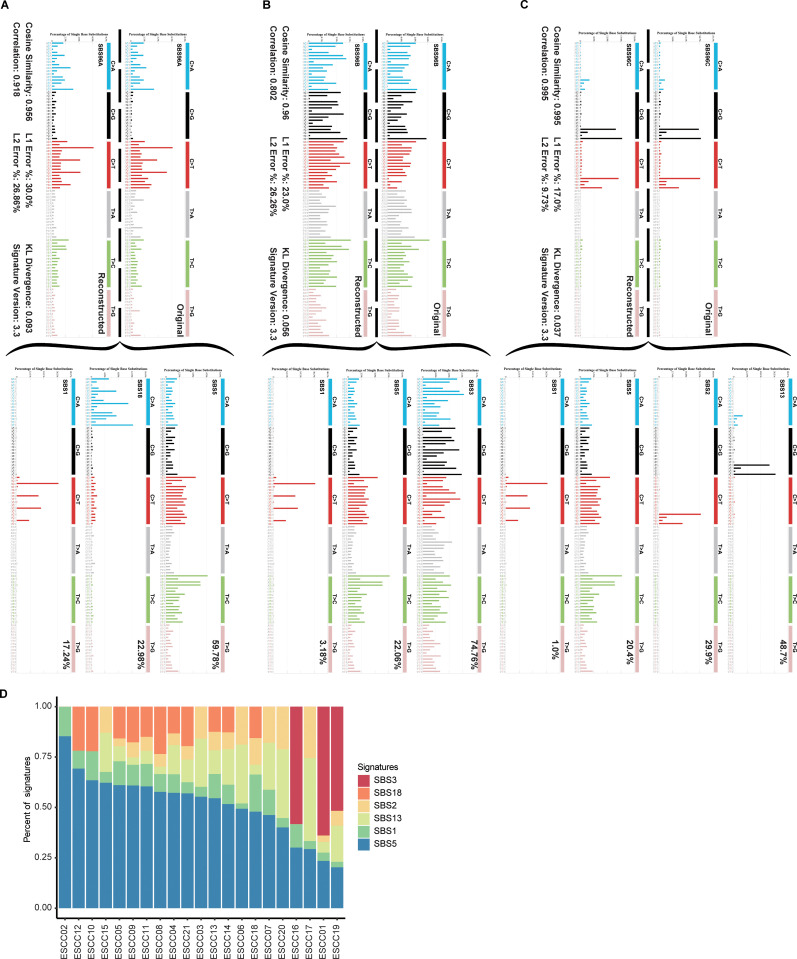
COSMIC Single base substitution signatures identified in non-hypermutated ESCC. (A) De novo identified signature SBS96A is matched to a combination of COSMIC signatures SBS5, SBS18 and SBS1. (B) De novo identified signature SBS96B is matched to a combination of COSMIC signatures SBS3, SBS5 and SBS1. (C) De novo identified signature SBS96C is matched to a combination of COSMIC signatures SBS13, SBS2, SBS5 and SBS1. (D) Frequency of decomposed COSMIC signatures in each sample.

Three SBS mutation signatures (SBS96A, SBS96B, and SBS96C) were identified based on the SBS in 96-element form, and these were composed of the COSMIC SBS1, SBS2, SBS3, SBS5, SBS13 and SBS18 mutational signatures (Fig 2). SBS96A was composed of the SBS5 (59.78%), SBS18 (22.98%), and SBS1 (17.24%) signatures (Fig 2A), whereas SBS96B was primarily composed of SBS3 (74.76%), SBS5 (22.06%), and SBS1 (3.18%) (Fig 2B). In addition, SBS96C mainly consisted of SBS13 (48.7%), SBS2 (29.9%), SBS5 (20.4%), and SBS1 (1.0%) (Fig 2C). Among these annotated mutational signatures, the majority of variants were associated with SBS5 (21/21) and SBS1 (21/21), while a subset of variants were associated with SBS2 (17/21), SBS13 (17/21), SBS18 (11/21), and SBS3 (3/21) (Fig 2D).

### Identification of copy number variations and structural variations

**Fig 3:**
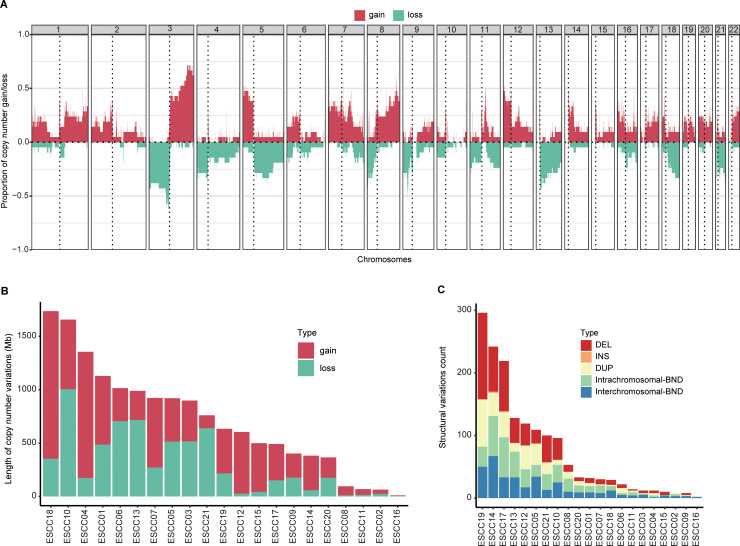
Copy number variation and structural variation classification. (A) Copy number gain and loss proportions in patients with non-hypermutated ESCC. (B) CNV lengths per sample. (C) The per-sample frequencies of different SV types.

For whole-genome analyses of somatic CNAs in ESCC tumor tissue samples, the frequency of somatic copy number gains and losses was examined at the cohort level (Fig 3). The regions that showed the most amplifications were located in chromosome arms 3q and 5p, while those with the most deletions where located in 3p (Fig 3A). The length of somatic CNAs for each sample pair in this cohort is shown in Fig 3B. In the 21 analyzed paired tumor and normal tissue samples, the average CNA length was about 714.05 Mb (range: 10.22 Mb – 1735.70 Mb), and the CNA length of ESCC18 and ESCC10 was more than 1500 Mb (Fig 3B). For further details regarding CNAs in this patient cohort, see S4 Table. Our study also identified a higher frequency of CNAs in chromosome 3q in TCGA-ESCA (S4A Fig). The total length of the genome segments affected by either gains or losses in patients with esophageal cancer in our ESCC dataset was significantly higher than data from TCGA-ESCA (S4B Fig).

Somatic SVs were also classified for these 21 ESCC patients. In total, 1,574 somatic SVs were detected, including 728 breakends, 530 deletions, 311 tandem duplications, and 5 insertions (S5 Table and Fig 3C). Based on the associations among chromosomes, these 728 breakends were further characterized as 381 and 347 intrachromosomal and interchromosomal breakends, respectively (Fig 3C). In addition, 493 SV events (31.32%) were predicted to impact exonic or splicing regions (275 deletions, 186 tandem duplications, and 32 breakends).

### Circular extrachromosomal DNA analyses

Computational methods were next used to analyze these WGS data in an effort to detect focal amplifications as a means of studying the ecDNA landscape of ESCC. Amplicons were defined as sets of connected genomic intervals with copy number amplifications, and amplicon structures were defined as ordered lists of segments from the amplicon intervals [9].

**Fig 4:**
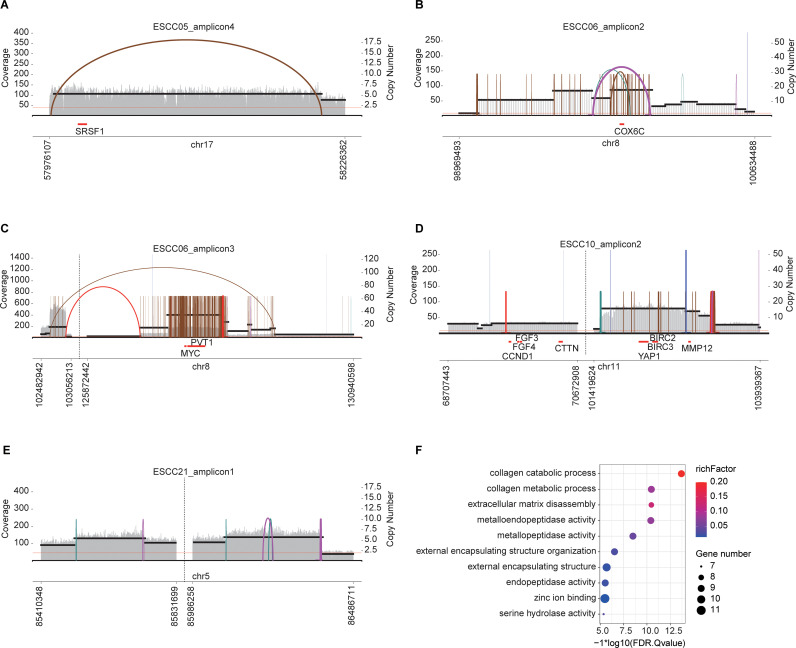
Rearrangement signature visualization for each amplicon containing ecDNA detected in patients with ESCC. (A-E) The SV view for different samples. Amplicon intervals are shown on the X-axis, while the depth of coverage for these intervals is indicated by the left Y-axis and vertical grey bars, and copy number estimations are indicated by the right Y-axis and black horizontal lines. Arcs represent discordant read pair clusters, and are color-coded as follows for read mapping orientation: red indicates length discordant in expected orientation (forward-reverse), brown indicates everted read pairs (reverse-forward), teal indicates that both reads map to the forward orientation, and magenta indicates that both reads map to the reverse orientation. Connections to the source vertex are represented using blue vertical lines. Oncogene annotations are shown in the bottom panel [9].(F) ecDNA genes enrichment analysis results.

In total, 29 amplicons were identified in 13 patients that could be classified as ecDNA, breakage-fusion-bridge (BFB), complex non-cyclic, and linear amplifications. Specifically, ecDNA was detected in patients ESCC05, ESCC06, ESCC10, and ESCC21 (S6 Table and Fig 4). As shown in S6 Table, these detected ecDNAs were annotated to genes *DYNLL2*, *EPX*, *COX6C*, *FBXO43*, *AZIN1*, *AZIN1-AS1*, *ANGPTL5*, *BIRC2*, *NBPF22P* and et al. In particular, *SRSF1*, *COX6C*, *MYC*, *PVT1*, *BIRC2*, *BIRC3*, *MMP12*, and *YAP1* were identified as ecDNA-related oncogenes (Fig 4A-D). Amplification events can induce oncogene activation, and oncogene expression is a key driver of tumorigenesis [10–13]. Significantly, most of the oncogenes within ecDNA had higher level expression in primary tumor compared to solid tissue normal in TCGA-ESCA (S5 Fig), which might indicate the importance of oncogenes within ecDNA in tumorigenesis.

**Fig 5:**
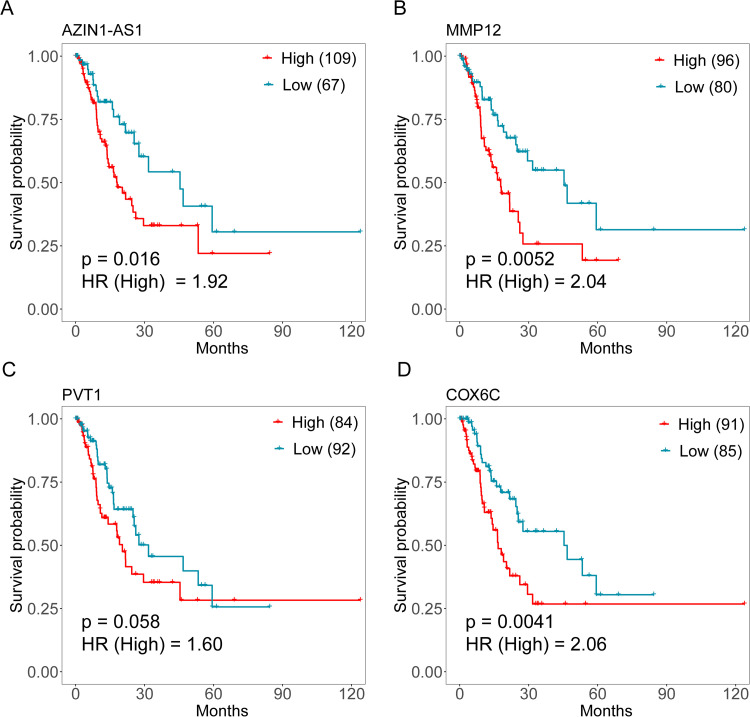
Survival analyses for ecDNA-associated genes in the TCGA-ESCA dataset. (A-D) TCGA-ESCA dataset (n = 176) was used to generate disease-free survival plots for patients grouped based on high and low expression values for the indicated genes.

Gene set enrichment analyses were additionally performed as a means of exploring the potential functions of these ecDNA genes. The top enriched terms associated with these genes included the following: collagen catabolic process, collagen metabolic process and extracellular matrix disassembly (Fig 4F). Possible relationships between ecDNA genes and disease-free survival (DFS) were also explored for patients in TCGA-ESCA database, revealing that patients expressing higher levels of *COX6C* (HR = 2.06, Logrank p = 0.0041), *AZIN1-AS*1 (HR = 1.92, Logrank p = 0.016), *MMP12* (HR = 2.04, Logrank p = 0.0052), and *PVT1* (HR = 1.60, Logrank p = 0.058) exhibited a shorter DFS duration (Fig 5A-D). High expression of *AZIN1-AS1*, *COX6C*, *MMP12* and *PVT1* might be associated with a worse survival outcome both in TCGA ESCC and esophageal adenocarcinoma (EAC) datasets (S6 Fig). And high expression of them significantly associated with poor disease-free survival (HR = 2.17, p-value = 0.003) after adjustment for age, gender and stage (S7 Table).

## Discussion

In the present study, a WGS approach was used to facilitate the comprehensive characterization of the genomic characteristics of ESCC. Mutational landscapes were examined for 21 patients with non-hypermutated ESCC, with a focus on SNVs and INDELs. In total, the most frequently mutated genes including *CSMD3*, *EP300*, *FAM135B*, *NOTCH1*, and *TP53* were identified in ESCC, consistent with previous studies in recent years [8,14–16]. These 5 genes were annotated in the COSMIC Cancer Gene Census and validated by TCGA-ESCA, which indicated these genes especially *TP53* and *NOTCH1* might play crucial roles in the tumorigenesis of ESCC. In addition, six COSMIC Mutational Signatures (SBS1, SBS2, SBS3, SBS5, SBS13, and SBS18) were detected in these analyses. And an average of approximately 714.05 Mb genome length per tumor samples harbored copy number gains (415.42 Mb) or losses (298.63 Mb). In total, 1,574 somatic SVs were detected in this ESCC cohort. Finally, 5 ecDNA events that included 8 ecDNA-related oncogenes in 4 patients were identified. High levels of the ecDNA-related genes *COX6C*, *AZIN1-AS1*, *PVT1*, and *MMP12* were also found to be associated with poorer DFS among patients in the TCGA-ESCA dataset.

Mutational signatures are invaluable tools that can aid efforts to understand the biological basis for oncogenesis [17], and several mutational signatures were successfully extracted in the present study. The SBS5 and SBS1 mutational signatures are correlated with aging-related factors [18], which was the predominant mutational process associated with ESCC incidence in this study cohort. SBS5 has also been shown to be correlated with tobacco smoking [19]. SBS2 and SBS13 have been identified as APOBEC (apolipoprotein B mRNA-editing enzyme, catalytic polypeptide-like)-related mutational signatures [20], while SBS18 was characterized as a reactive oxygen species-induced defective base excision repair-related signature [19,21]. The SBS3 COSMIC signature was also related to DNA damage repair [22,23]. SBS1 and SBS5 were detected in 21 ESCC patients in this study cohort, while SBS2 and SBS13 were evident in over half of these patients. This suggests that with the exception of age-related factors, APOBEC-related mutational signatures may be a key factor associated with ESCC development and progression. In a previous study of ESCC whole exon sequencing in a Chinese population, APOBEC-associated mutation signatures (including COSMIC signature 2 and 13) were found to be significantly enriched in some patients, and patients subtypes carrying APOBEC signatures showed higher overall survival [24]. A positive APOBEC mutation profile in patients with ovarian clear cell carcinoma is associated with higher lymphocyte infiltration and better prognosis [25]. The APOBEC mutational signature can also be used as a potential predictor of immunotherapy response in NSCLC [26]. In addition, the related mutational signature of other APOBEC family members also play a role in different cancers, for example, APOBEC3s can produce mutational signature 2 and 13 in COSMIC under the wrong replication and repair pathways, and is involved in cervical cancer, head and neck squamous cell carcinoma, and bladder cancer [27]. This is consistent with prior results demonstrating that APOBEC activation is essential for ESCC development [3].

Circular extrachromosomal DNA enables accelerated tumor evolution through mechanisms distinct from traditional chromosomal inheritance, contributing to high levels of oncogene amplification, intratumoral heterogeneity, and therapeutic resistance [28–30]. Prior research has suggested that ecDNA may be a contributor to the amplification of various oncogenes and immunomodulatory genes in the context of esophageal adenocarcinoma development [31]. Here, 8 ecDNA-related oncogenes were identified (*SRSF1*, *COX6C*, *MYC*, *PVT1*, *BIRC2*, *BIRC3*, *MMP12*, and *YAP1*), of which two (*MYC* and *BIRC2*) are candidate ecDNA-related oncogenes previously reported in studies of ESCC conducted by Cui et al. [32]. Research focused on Barrett’s esophagus and esophageal adenocarcinoma have also suggested that *BIRC2*, *BIRC3*, *MMP12*, *MYC*, *PVT1*, and *YAP1* may be ecDNA-related oncogenes [31]. Kim et al. [33] further found that patients who suffer from ecDNA events tend to exhibit poor survival outcomes across a variety of cancer types. In the present study, ESCC patients in the TCGA-ESCA cohort with tumors overexpressing *COX6C*, *AZIN1-AS1*, *PVT1*, and *MMP12* might to be associated with poor disease-free survival in TCGA-ESCA patients. *COX6C*, *PVT1* and *MMP12* were identified as oncogene. Notably, *AZIN1-AS1* has been shown to serve as a novel oncogenic long non-coding RNA capable of promoting non-small cell lung cancer progression [34].To the best of our knowledge, there were limited ESCC WGS studies focused on ecDNA. Significantly, our work found that *COX6C*, *PVT1*, *MMP12* and *AZIN1-AS1* within ecDNA might be associated with poor survival. And most of ecDNA-related oncogenes identified in our study had higher level expression in primary tumor compared to solid tissue normal in TCGA-ESCA, which might indicate the importance of oncogenes within ecDNA in tumorigenesis of ESCC and provides insights into the selection of biomarkers of different type of esophageal cancer.

In summary, the present results highlight the mutational landscape of ESCC, providing novel insight into the genomic changes associated with this cancer type. The present results may aid in the future investigation of biomarkers that can guide the diagnosis of ESCC of the identification of new targets amenable to therapeutic intervention. However, this study is subject to certain limitations, such as the limitation of sample size and sequencing depth, and future large-scale studies and clinical analyses focused on patient survival outcomes will be essential for the effective experimental validation of these findings.

## Materials and methods

### Patients and sample collection

The Ethics Committee of Liaocheng People’s Hospital approved this study, and all patients provided informed consent prior to sample collection. Paired primary tumor and normal tissue samples were harvested from 22 ESCC patients in the Liaocheng People’s Hospital between August 2018 and March 2019. All pathological diagnoses were confirmed by pathologists in accordance with WHO criteria, and the TNM staging system established by the American Joint Committee on Cancer was used for tumor staging. Detailed patient characteristics are presented in S1 Table. The written informed consent was obtained from the participating subject. The experimental procedures conformed to the guidelines approved by the Ethics Committee of Liaocheng People’s Hospital and The Institutional Review Board BGI (BGI-IRB) on bioethics and biosafety. This study was conducted according to the guidelines of the Declaration of Helsinki and approved by the Ethics Committee of Liaocheng People’s Hospital (2018043, 17 May 2018).

### Whole genome sequencing

The QIAamp DNA Mini Kit (Qiagen, Germany) was used based on provided directions to extract genomic DNA from patient tumor and normal tissue samples. DNA sample concentrations were measured with a Qubit Fluorometer (Thermo Fisher Scientific, USA), and DNA quality was analyzed via agarose gel electrophoresis. WGS library construction was performed as in prior studies [35], after which 100 bp paired-end sequencing was performed with a DIPSEQ-T1 sequencer (MGI, China).

### Data processing and reads mapping

SOAPnuke [36] v2.0 was used to filter the raw reads, removing any reads containing >10% N bases, reads containing sequence adaptors, and any low-quality reads (quality < 5) with > 50% in one read. Clean reads were then aligned to the hg38 human reference genome using the BWA-MEM [37] v0.7.17-r1188 algorithm with default parameters, after which aligned reads were sorted with Picard [38] v2.18.27, and PCR duplicates were marked. The Genomic Analysis Toolkit (GATK) [39] v3.8-1 was then used for base quality score recalibration and local realignment as described in the GATK best practice guidelines [40], and the GATK ContEst [41] module was used to delete samples with a contamination level > 1%. Tumor and normal samples from the same biological source were then matched with bam-matcher [42], and high-quality alignment results were used to call somatic variations in downstream analyses.

### Somatic mutation and mutation signature identification

Somatic SNV and INDEL detection was performed with the Genome Analysis Toolkit (GATK) [39] v4.2.6.1 Mutect2 and FilterMutectCalls with default parameters. Initially, the FilterMutectCalls function was used to filter all somatic mutations detected by Mutect2, after which those somatic SNVs marked with ‘PASS’ by FilterMutectCalls were filtered to identify those variants with a sequencing depth ≥ 10, ≥ 4 supported reads in tumors, and ≤ 2 supported reads in paired normal samples. Somatic INDELs marked with ‘PASS’ by FilterMutectCalls were additionally filtered to identify those variants with a sequencing depth ≥ 10, ≥ 5 supported reads in tumors, and ≤ 1 supported reads in paired normal samples. Identified somatic SNVs and INDELs were then annotated using ANNOVAR with refGene [43]. Finally, maftools [44] was used to converts and visualize the results of annovar annotations. Meanwhile, somatic mutations (Masked Somatic Mutation) files of 179 primary tumors were also downloaded and processed from GDC TCGA-ESCA dataset using TCGAbiolinks [45] and maftools [44]. We used 38 Mb as the estimate of the exome size, and mutation burdern of each sample is equal to the total mutations/38 in TCGA-ESCA.

Mutational signatures were estimated using SigProfiler. The de novo extraction of mutational signatures from VCF files was performed using SigProfilerExtractor [46] v1.1.20, and COSMIC [47] signatures were used to annotate these de novo extracted signatures. Single base substitution (SBS) signatures identified using 96 different contexts were used for these analyses.

### Detection of copy number variations and structural variations

Somatic copy number alterations and tumor ploidy were estimated for each sample using Sequenza [48] v3.0.0. First, we used the mpileup function from SAMtools [49]v1.9 to convert BAM files into Pileup format, filtering for base quality ≥ 20 and mapping quality ≥ 20. Second, paired tumor and normal Pileup files were processed using the bam2seqz and seqz_binning modules from sequenza-utils with default parameters. The output from sequenza-utils was then further analyzed using the Sequenza R package to generate segmented copy number data. Copy number data for each segment obtained from Sequenza (except chrX and chrY) were divided by mean sample ploidy (CNt/Ploidy, S3 Table). The cnFreq function in the GenVisR [50] v1.29.3 R package was then used for the identification of copy number gains (2.5/2) or losses (1.5/2). Manta [51] v1.6.0 was used to call structural variations, and somatic SVs marked with ‘PASS’ by Manta when using the default parameters were retained for further analysis. Then, the Manta VCF files were converted to BEDPE formats using svtools [52]. All identified CNAs and SVs were subjected to refGene annotation using ANNOVAR [43]. Meanwhile, copy number (Masked Copy Number Segment) files of 179 primary tumors were also downloaded from GDC TCGA-ESCA dataset using TCGAbiolinks [45].

### Circular extrachromosomal DNA analysis

Circular extrachromosomal DNA detection was performed using the AmpliconArchitect [9] v1.3.r3 and the CNVkit [53] v0.9.9 following the AmpliconSuite-pipeline (https://github.com/AmpliconSuite/AmpliconSuite-pipeline). WGS data were used as inputs for AmpliconArchitect, which explores sources of focal amplification including circular ecDNA and breakage-fusion-bridge cycles in cancer-associated genomes. Outputs from this tool were classified using AmpliconClassifier [31] v0.4.13, and all analyses were performed based on the PrepareAA v0.1344.4 pipeline’s --run_AA and --run_AC functions with default parameters.

### Enrichment analysis and PPI network construction

Gene functional enrichment analyses were performed with the Gene Set Enrichment Analysis (GSEA) web server [54,55], and included analyses of Gene Ontology (GO) gene sets including the GO biological process, cellular component, and molecular function sets from the Human Molecular Signatures Database (MSigDB) [55–57]. The protein-protein interaction (PPI) network analysis was performed using the STRING database (Search Tool for the Retrieval of Interacting Genes/Proteins) [58] v12.0, an online tool and database of protein-protein interaction. A minimum required interaction score > 0.4 were selected and reconstructed in Cytoscape [59]v3.10.3, and cytoHubba plugin [60]v0.1 was used to find hub genes in PPI network. The top three genes with the highest prediction scores calculated by the Maximal Clique Centrality (MCC) algorithm were defined as the hub genes.

### Survival analysis

R packages survival and survminer were used to conduct survival analyses [61,62], with a focus on disease-free survival in the TCGA-ESCA dataset, using surv_cutpoint functions determine the optimal cutpoint as the cut-off for patient stratification. To further investigate the results, expression information (UCSC Toil RNA-seq Recompute) of 176 primary tumor samples and corresponding clinical data (TCGA Esophageal Cancer) including age, gender, survival time and pathologic stage were download from UCSC Xena project. R package survival [62] was used to perform multivariate cox regression analysis. All analyses are based on publicly published software, and the corresponding authors can be contacted for any additional data code requirements.

## Consent to publish

All authors have their consent to publish their work.

## Supporting information

S1 Table Clinical and pathological features for 22 ESCC patients. (XLSX)

S2 Table Details regarding somatic SNVs and INDELs. (XLSX)

S3 Table Gene set enrichment analysis. (XLSX)

S4 Table ESCC patients copy number variation data. (XLSX)

S5 Table Structural variations detected in ESCC patients. (XLSX)

S6 Table Focal amplification events detected in ESCC patients. (XLSX)

S7 Table Multivariate Cox regression analysis for disease-free survival in TCGA-ESCA. (XLSX)

S1 Fig PPI network analysis and hub genes screen. The hub gene nodes were highlighted in red.
(TIF)

S2 Fig Comparison of expression values of top mutated genes between primary tumor and solid tissue normal in TCGA-ESCA. Box plots expression values of TP53, NOTCH1, EP300, FAM135B and CSMD3 by different group.
(TIF)

S3 Fig Comparison of somatic mutations between ESCC and TCGA-ESCA. (A) Oncoplot for the top 30 mutated genes identified in patients with non-hypermutated TCGA-ESCA. (B) Comparison of somatic mutations per megabase of analyzed genomic sequence (mutation burden) between our ESCC study and TCGA-ESCA.
(TIF)

S4 Fig Comparison of copy number variations between ESCC and TCGA-ESCA. (A) Copy number gain and loss proportions in patients with non-hypermutated TCGA-ESCA. (B) Comparison of lenth of total copy number gain/loss between our ESCC study and TCGA-ESCA.
(TIF)

S5 Fig Comparison of expression values of ecDNA-related oncogenes between primary tumor and solid tissue normal in TCGA-ESCA. Box plots expression values of MYC,SRSF1,MMP12,PVT1,BIRC2,COX6C,BIRC3 and YAP1 by different group.
(TIF)

S6 Fig Survival analyses for ecDNA-associated genes in the TCGA-ESCA dataset. TCGA ESCC (n = 91) and TCGA EAC datasets (n = 85) were used to generate disease-free survival plots.
(TIF)
