# Supplementary figures and images for: Whole genome sequencing revealed esophageal squamous cell carcinoma related biomarkers
Source: PLoS One. 2025 Jun 26;20(6):e0323915. doi: 10.1371/journal.pone.0323915 (PMC12200652; doi:10.1371/journal.pone.0323915)

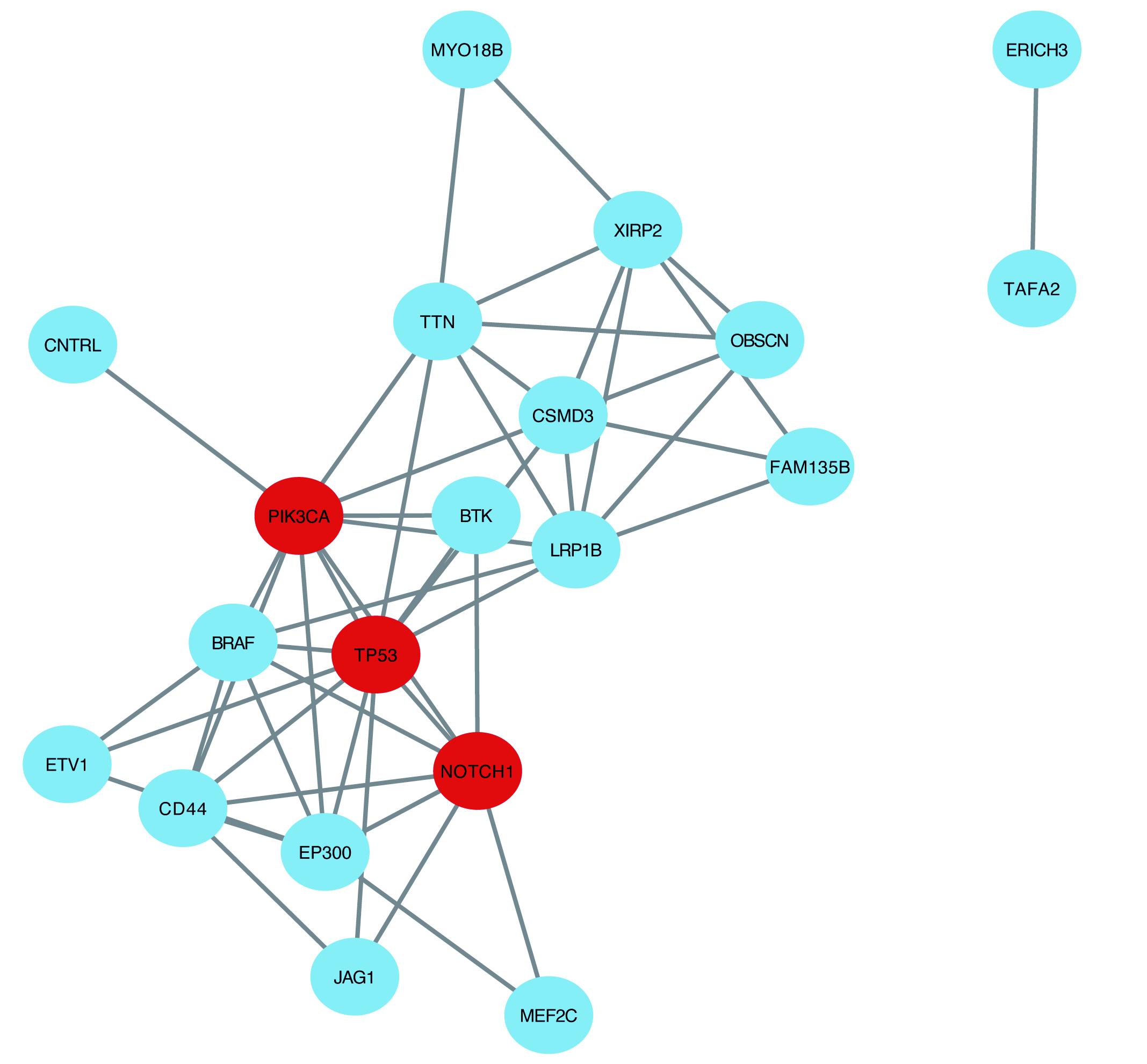

Supplement: S1 Fig — The hub gene nodes were highlighted in red. (TIF) [file pone.0323915.s008.tif]

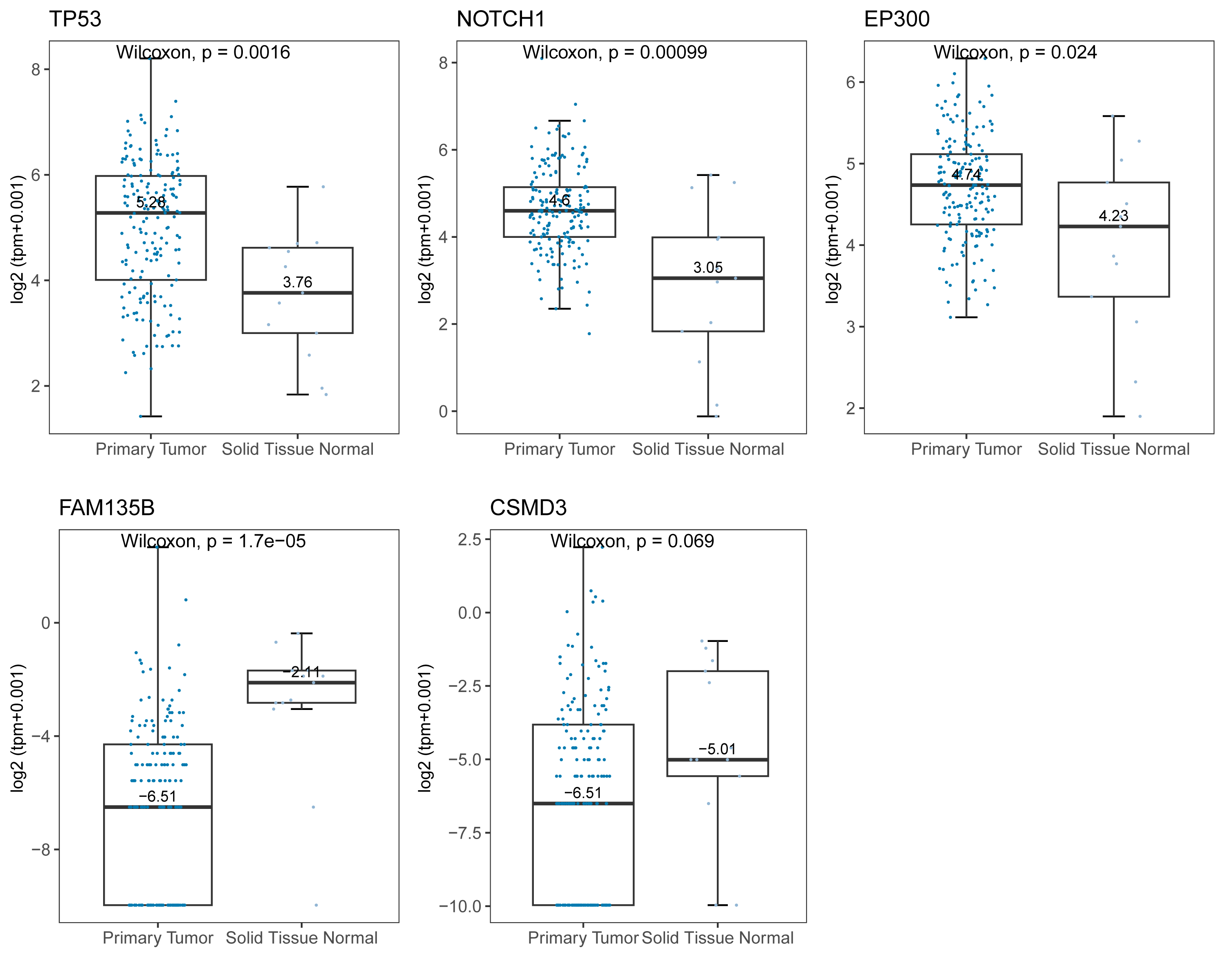

Supplement: S2 Fig — Box plots expression values of TP53, NOTCH1, EP300, FAM135B and CSMD3 by different group. (TIF) [file pone.0323915.s009.tif]

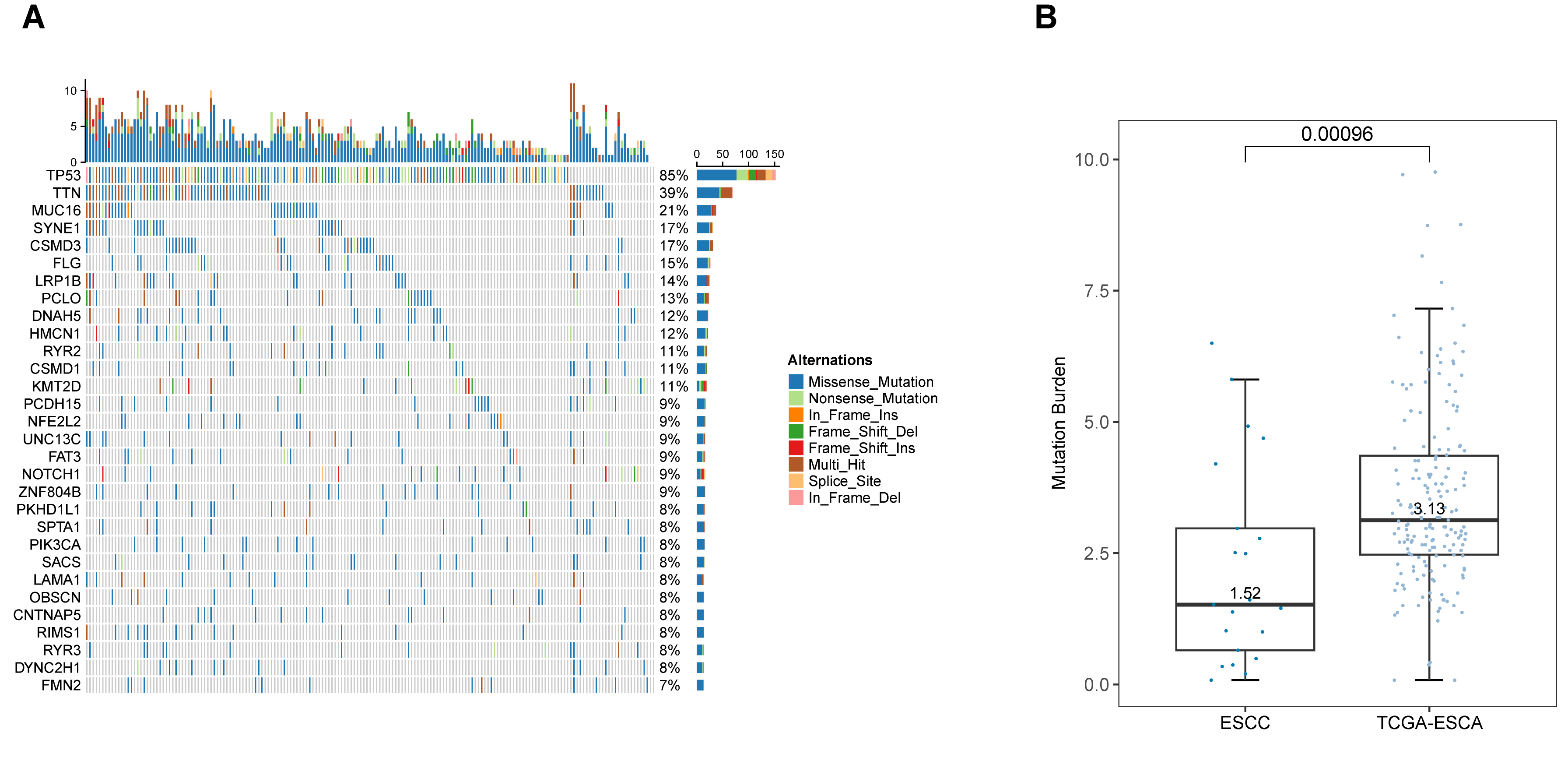

Supplement: S3 Fig — (A) Oncoplot for the top 30 mutated genes identified in patients with non-hypermutated TCGA-ESCA. (B) Comparison of somatic mutations per megabase of analyzed genomic sequence (mutation burden) between our ESCC study and TCGA-ESCA. (TIF) [file pone.0323915.s010.tif]

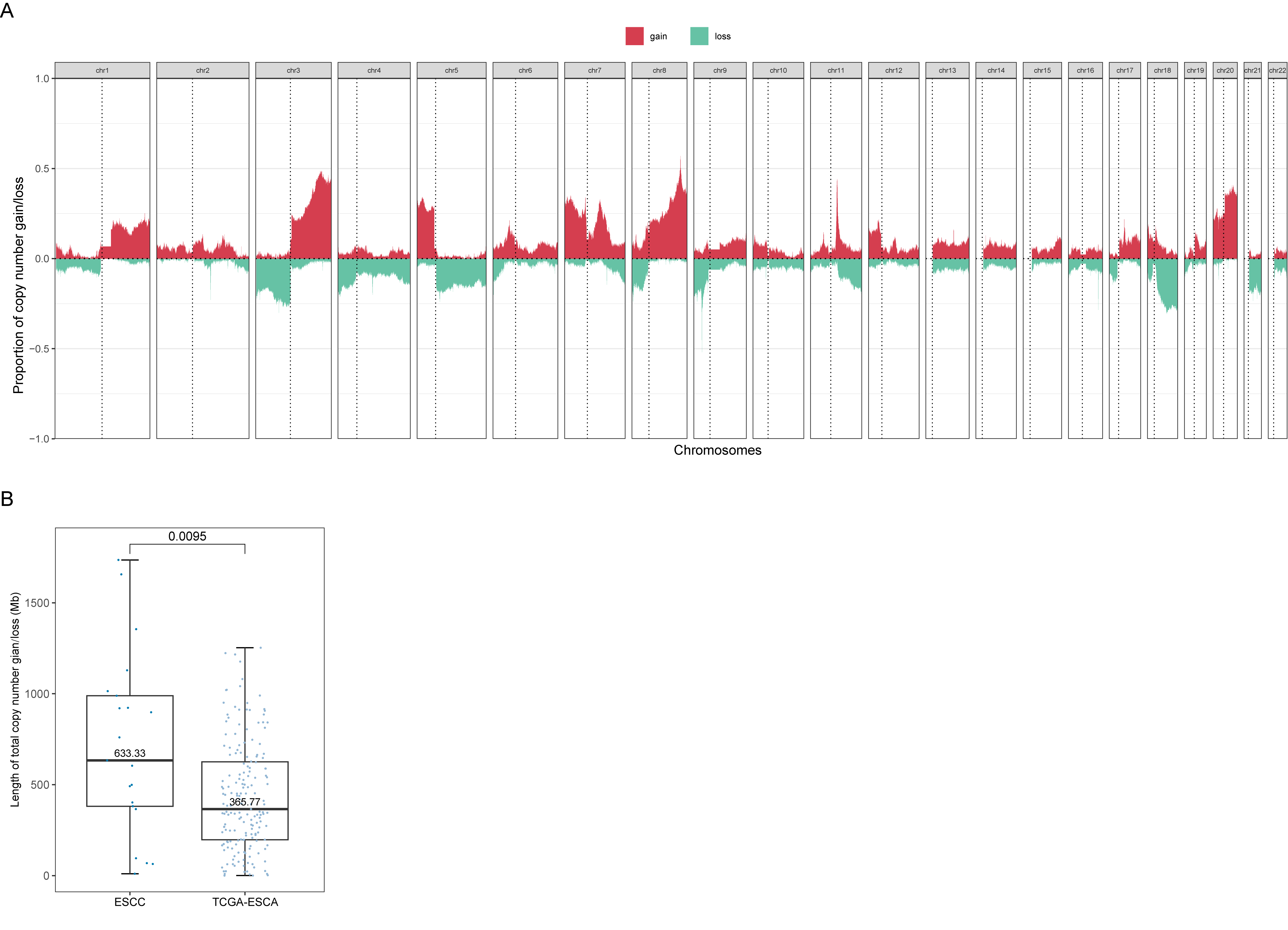

Supplement: S4 Fig — (A) Copy number gain and loss proportions in patients with non-hypermutated TCGA-ESCA. (B) Comparison of lenth of total copy number gain/loss between our ESCC study and TCGA-ESCA. (TIF) [file pone.0323915.s011.tif]

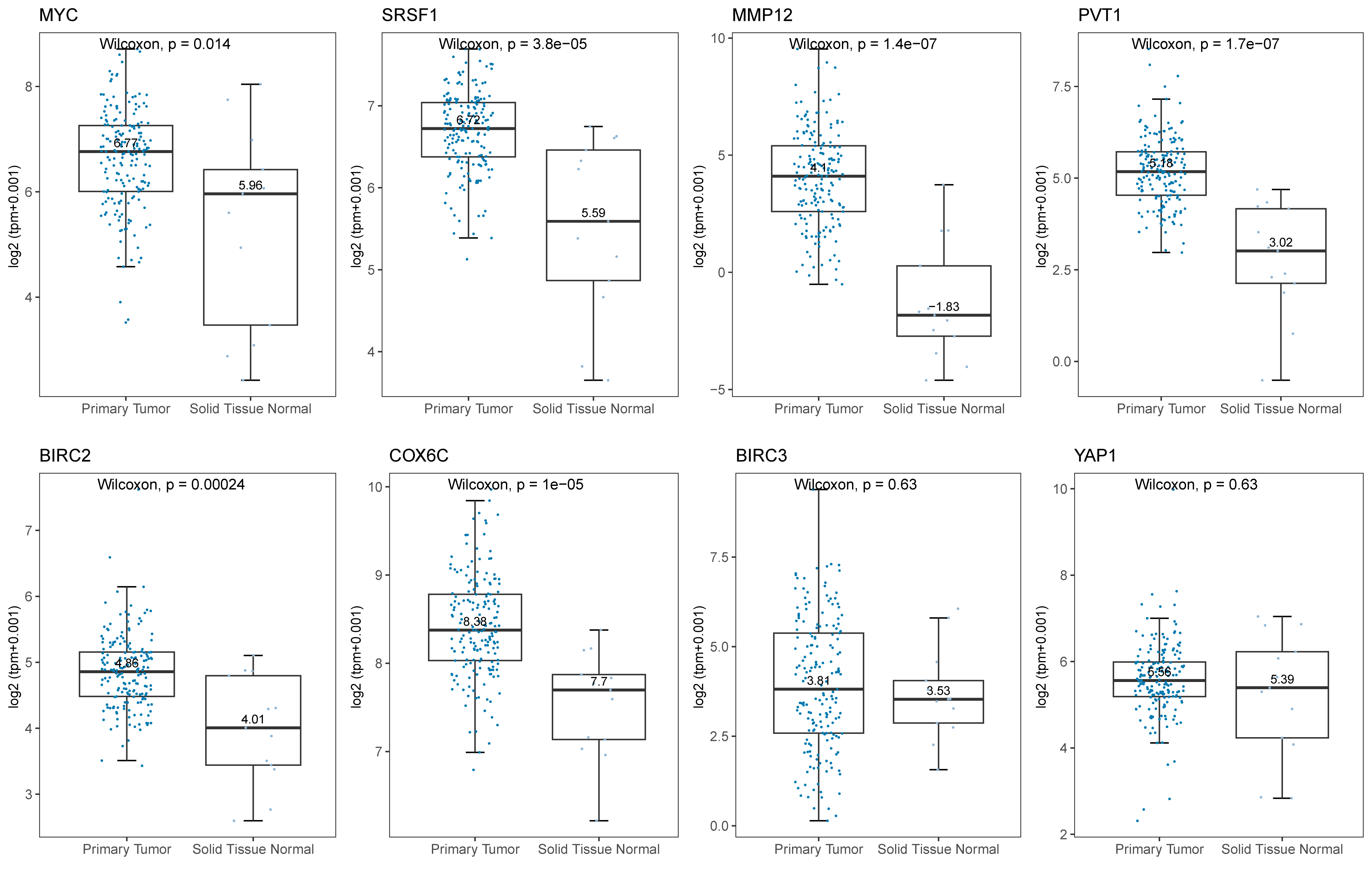

Supplement: S5 Fig — Box plots expression values of MYC,SRSF1,MMP12,PVT1,BIRC2,COX6C,BIRC3 and YAP1 by different group. (TIF) [file pone.0323915.s012.tif]
